# Herpes Zoster and Immunogenicity and Safety of Zoster Vaccines in Transplant Patients: A Narrative Review of the Literature

**DOI:** 10.3389/fimmu.2018.01632

**Published:** 2018-07-16

**Authors:** Lei Wang, Erik A. M. Verschuuren, Coretta C. van Leer-Buter, Stephan J. L. Bakker, Anoek A. E. de Joode, Johanna Westra, Nicolaas A. Bos

**Affiliations:** ^1^Department of Rheumatology and Clinical Immunology, University Medical Center Groningen, University of Groningen, Groningen, Netherlands; ^2^Department of Pulmonary Diseases, University Medical Center Groningen, University of Groningen, Groningen, Netherlands; ^3^Department of Medical Microbiology, Division of Clinical Virology, University Medical Center Groningen, University of Groningen, Groningen, Netherlands; ^4^Department of Internal Medicine, Division of Nephrology, University Medical Center Groningen, University of Groningen, Groningen, Netherlands

**Keywords:** varicella zoster virus, herpes zoster, postherpetic neuralgia, transplantation immunology, herpes zoster vaccine

## Abstract

This narrative review focuses on the herpes zoster (HZ) and its prevention in transplant patients. Varicella zoster virus (VZV) is highly contagious and distributed worldwide in humans. Primary VZV infection usually causes varicella and then establishes a lifelong latency in dorsal root ganglia. Reactivation of VZV leads to HZ and related complications such as postherpetic neuralgia. Age and decreased immunity against VZV are important risk factors for developing HZ. Transplant patients are at increased risk for developing HZ and related complications due to their immunocompromised status and the need for lifetime immunosuppression. Diagnosis of HZ in transplant patients is often clinically difficult, and VZV-specific antibodies should be determined by serologic testing to document prior exposure to VZV during their pre-transplant evaluation process. Although antiviral agents are available, vaccination should be recommended for preventing HZ in transplant patients considering their complicated condition and weak organ function. Currently, there are two licensed HZ vaccines, of which one is a live-attenuated vaccine and the other is a HZ subunit vaccine. Both vaccines have shown promising safety and efficacy in transplants patients and especially the subunit vaccine could be administered post-transplant since this vaccine does not contain any live virus. Larger studies are needed about safety and immunogenicity of HZ vaccines in transplant populations, and extra efforts are needed to increase vaccine usage according to guidelines.

## Introduction

Varicella zoster virus (VZV) belongs to the Alphaherpesvirinae subfamily and is a member of the Varicellovirus genus. VZV is highly contagious and distributed worldwide in humans (1). Primary VZV infection usually occurs during childhood and causes varicella (chickenpox). Following infection by contact with aerosolized vesicle fluid or through the respiratory route, VZV first replicates in epithelial cells of the upper respiratory mucosa. A disseminated vesicular rash appears subsequently after an incubation period of approximately 10–21 days. After primary infection, immunity to VZV is established, while the virus travels by retrograde spread along sensory neurons to the trigeminal and dorsal root ganglia establishing a lifelong latency (2). In elderly people or immunocompromised patients, a reduction in the ability of an appropriate immune response could lead to reactivation of VZV from latency allowing the virus to travel antegradely from the sensory ganglia to the skin nerve terminals and spreading to skin epithelial cells leading to the clinical signs of herpes zoster (HZ) (1). Although the symptoms of HZ normally resolve within 2–4 weeks, about 10% of patients develop postherpetic neuralgia (PHN)—the most frequent chronic complication of HZ. PHN is defined as pain persisting more than 3 months after the onset of the rash in the same affected area. It can interfere with the patients’ sleep and daily activities, causing a major loss of quality of life (3). In a systematic review including 130 studies conducted in 26 countries, the incidence rate of HZ was calculated at 3–5 per 1,000 person-years in North America, Europe, and Asia-Pacific, with a notable rise in persons over 50 years of age, reaching 8–12 per 1,000 person-years at age 80 years. The authors also reviewed the risk of developing PHN in patients with HZ and found it to range between 5 and 30%, with more than 30% of patients with PHN experiencing persistent pain for more than 1 year (4). Age is therefore an important risk factor for HZ. Several studies suggested increasing trends of HZ incidence over the past decade, which also lead to an increase in the prevalence of PHN, but the reasons for these trends are currently still unknown (5). Even more severe complications such as disseminated zoster occur mainly in immunocompromised patients. Disseminated HZ is usually characterized by vesicles spreading beyond the distribution of the affected dermatome, with the potential to affect other organs than the skin, potentially leading to pneumonia, encephalitis, and hepatitis with a 5–10% fatality rate (6).

Primary VZV infection induces both humoral and cellular immune responses. Several studies suggested that humoral immunity appears later and plays a less prominent role in bridling primary VZV infections compared with cellular immunity. This is supported by the fact that children with B cell deficiencies often recover uncomplicated from primary varicella infections, while children with T cell deficiencies are at high risk of progressive varicella (7). With regard to prevention of VZV reactivation, cell-mediated immunity (CMI) is believed to be more important than antibodies, although the mechanisms of protection are not fully understood. This is in line with the fact that the numbers of VZV-specific T cells decrease, whereas anti-VZV-immune globulin G (IgG) titers remain relatively stable over time (8, 9). Another study showed that in the first week after HZ rash onset, greater VZV CMI responses correlated with lower severity of disease, while VZV antibody responses did not correlate with severity of HZ or PHN (10). Type I interferons (IFNs) including IFN-α and IFN-β dominate the innate defense system against virus infections. During VZV replication in the skin after reactivation, the signal transducer and activator of transcription 3 is activated by VZV in infected cells, thereby suppressing the expression of INF-α and STAT1, whereas surrounding uninfected cells show upregulation of IFNs and STAT1 (2). Besides, type I and type III IFNs can induce the Janus kinase-signal transducer and activator of transcription (JAK/STAT) pathway, which leads to phosphorylation of STAT1 and STAT2 followed by IFN-stimulated genes expression. Verweij et al. demonstrated that VZV was able to inhibit type I IFN-activated signal transduction by degrading IFN regulatory factor 9 and inhibiting STAT2 phosphorylation of the JAK–STAT pathway (11). Due to the lack of robust models for latency, the mechanisms for VZV latency and subsequent reactivation are still being poorly understood. Sadaoka et al. established an *in vitro* system which recapitulates elements of VZV latency and reactivation *in vivo* (12). C-Jun N-terminal kinase (JNK) pathway was found to play a critical role in the viral reactivation using this system and pharmacologic blockade of the JNK pathway could inhibit VZV gene expression, lytic replication, and reactivation (13). A better understanding of the mechanisms underlying VZV reactivation and infection, and how the adaptive immune system can effectively protect during this process, is still largely unknown and an important topic for future investigations.

Transplantation is the only curative therapeutic option for terminal organ failure. In recent years, the number of solid organ transplant (SOT) and hematopoietic stem cell transplant (HSCT) recipients is continuously increasing. For example, about 29,000 SOTs in the U.S. and 19,000 HSCTs worldwide are performed annually (14). The development of novel immunosuppressive agents and better diagnosis of graft rejection have dramatically improved survival of transplant patients (15). Transplant patients are, however, at increased risk for developing HZ and related complications due to the need for lifetime immunosuppression. The type of transplantation, immunosuppression, and antiviral prophylaxis may influence HZ incidence. According to several trials and retrospective studies published in the last decade, incidence of HZ varied between 3.5 and 9% in kidney (16–20), 6.9 and 7.2% in liver (18, 21), 11.6 and 14.3% in lung (22–24), and 16.2 and 16.3% in heart (18, 25) transplant recipients (Table 1). Overall, liver transplant recipients have a lower incidence of HZ, possibly due to their relatively resistance to rejection and less requirement of immunosuppression compared with other organ recipients, whereas heart and lung transplants had a high rate of HZ possibly explained by the high doses of continuous immunosuppression (18, 26).

**Table 1:** The incidence of herpes zoster (HZ) in transplant patients.

Reference	Type of transplantation	No. of patients	No. of patients who developed HZ	Crude incidence of HZ (%)	Calculated incidence of HZ (cases/1,000 person-years)
(16)	Kidney	612	37	6	28
(17)	Kidney	1,139	40	3.5	^a^
(18)	Kidney	500	45	9	24.4
	Liver	461	32	6.9	18.3
	Heart	80	13	16.3	40
(19)	Kidney	450	29	6.4	20.6
(20)	Kidney	444	35	7.9	^a^
(21)	Liver	377	27	7.2	17.83
(22)	Lung	239	29	12.1	55.1
(23)	Lung	198	23	11.6	^a^
(24)	Lung	119	17	14.3	38.2
(25)	Heart	314	51	16.2	31.6

*^a^The reference did not show this data*.

**Figure 1:**
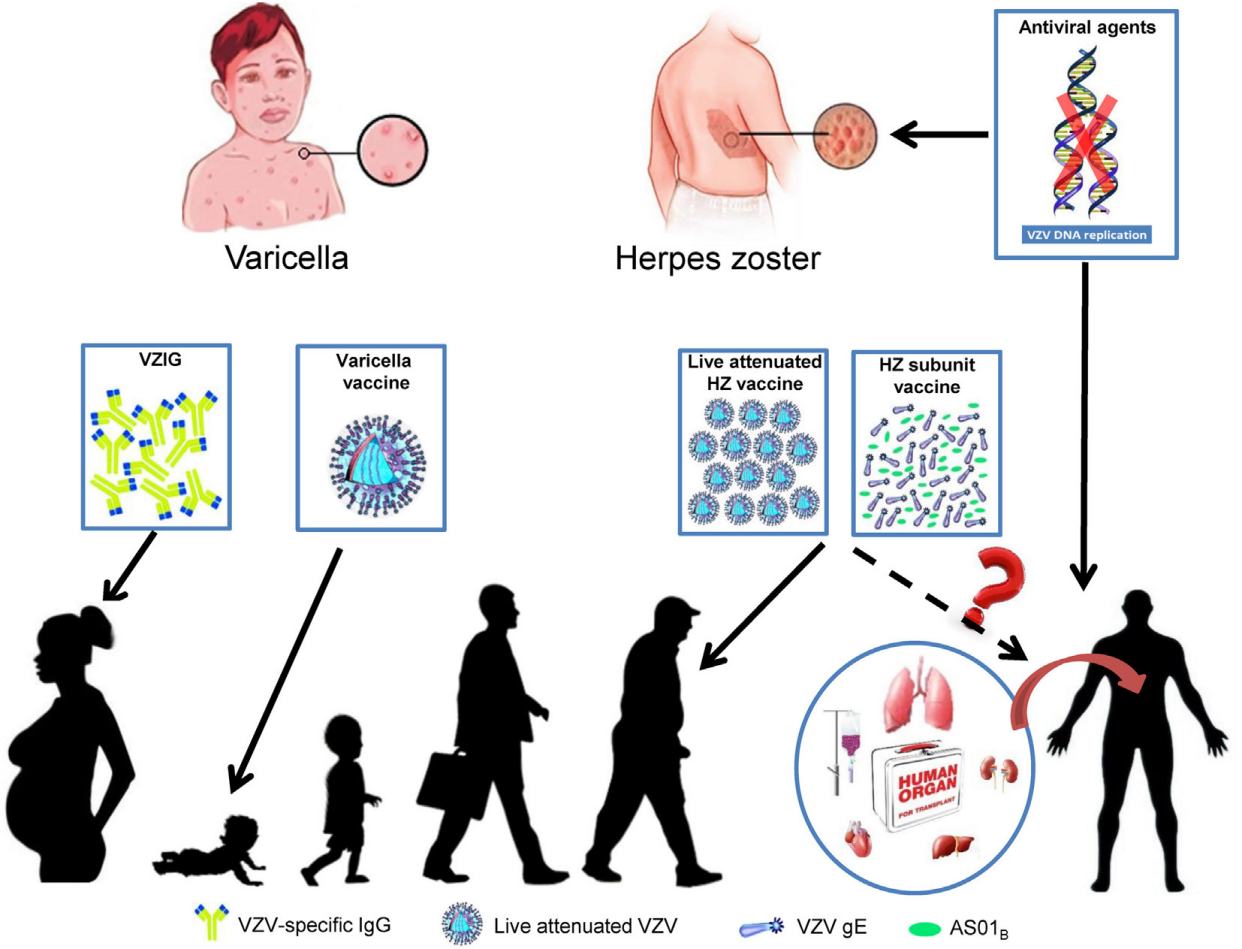
Treatments and prevention of varicella and herpes zoster (HZ).

In this article, we have performed a narrative review of the literature in recent years, providing an in-depth analysis of the development of diagnosis, treatment, and prevention of HZ, and retrospected efficacy and safety of the two currently licensed zoster vaccines in immunocompromised adults, with a focus on transplant patients (Figure 1). This paper aims to emphasize the dangerous situation of transplant patients facing HZ and the importance of future clinical trials about safety and efficacy of zoster vaccines in these HZ high-risk populations.

## Diagnosis

Generally, both primary varicella and HZ have typical clinical presentations that allow for a presumptive clinical diagnosis (26). Primary varicella rash (initially macular, proceeding to fluid-filled vesicles then turning yellow pustular) occurs on the trunk and face, and spreads to involve much of the skin surface (27). The diagnosis of HZ is based on the characteristic cutaneous eruption, which always presents as erythematous vesicular rash following dermatomal distribution with localized neurological pain (26).

Diagnosis of HZ in transplant patients is clinically often difficult, because HZ in these patients is more likely to present as atypical mucocutaneous forms that could mimic other cutaneous diseases, such as herpes simplex virus (HSV) infection and drug reactions (28), and may present with multiorgan involvement. It rarely develops into invasive complications with delayed or absent rash (26). In case of diagnostic uncertainty, laboratory testing [polymerase chain reaction (PCR)] is suggested and is becoming the standard for confirmation in transplant patients (29).

Viral culture and direct immunofluorescent assay (DFA) are the conventional methods for detecting VZV and have long been considered the gold standards. However, both methods have limitations such as time-consuming, insensitivity, and lack of standardization (30), and DFA also requires great technical expertise and has a high probability to mistakes in specimen collection and storage conditions (31).

Polymerase chain reaction is the most sensitive test for VZV and different PCR-based methods have been introduced to the clinical laboratory, for example, conventional PCR, nested PCR, and real-time PCR (32). PCR can detect VZV virus in vesicle fluid swab, biopsies, cerebrospinal fluid (CSF), intraocular fluids, and blood samples (29). Among these, multiplex real-time PCR assays can simultaneously detect VZV and HSV in CSF and lesion swab specimens in a single test (33). The multiplex assay provides accurate and rapid diagnostic capabilities for the diagnosis and differentiation of HSV and VZV infections with lower costs (34).

In all transplant patients, VZV-specific antibody levels should be determined by serologic testing to document prior exposure to VZV during their pre-transplant evaluation process. The VZV-specific antibodies level can be used for evaluation of the post-transplant risk of seronegative patients developing chickenpox and seropositive patients developing HZ (26). Determination of an increase in anti-VZV IgG and IgM and measurement of VZV DNA in CSF or peripheral blood by PCR are the methods to confirm diagnosis of HZ sine herpete (defined as dermatomal distribution pain occurring without an antecedent rash) (28, 29). Antibody tests for VZV include the fluorescent antibody to membrane antigen (FAMA), enzyme immunoassay, and enzyme-linked immunosorbent assays (ELISA). These tests use whole antigens of the virus or antigen extracted from VZV-infected cell cultures, whereas other tests use the external glycoproteins [glycoprotein E (gE), gB, and gH] as antigens, such as the gpELISA (35). FAMA is highly sensitive and considered the gold standard test for VZV antibodies, but is labor intensive and not automated (36). ELISA is widely used because of its simplicity and the possibility of automation, but still lacks sufficient sensitivity of detecting vaccine seroconversion (37). Combination of serology testing and real-time PCR on paired serum and CSF/intraocular fluid can be used for diagnosing HZ with cerebral and ocular complications (38). It is important to develop new precise assays because false-negative and false-positive results due to limitations of serologic testing may lead to unnecessary vaccinations or therapy (35). Even more important is the limitation that antibody titers do not necessarily correlate with protection. It is unknown whether high antibody titers are correct correlates of VZV protection or only a measure of past infection. Seemingly protective antibody titer in a whole virus ELISA may not correlate with protective levels of antibodies directed against membrane antigens (39). Development of methods to determine potential HZ-predisposing VZV-specific cellular adaptive immunity could be promising for the future, because VZV-specific antibodies only have an apparent limited protective effect (24).

## Treatment

### Herpes Zoster

The primary goals for the treatment of HZ by taking antiviral agents are to control the replication of VZV in infected cells and to soothe and protect the infected skin. Lowering the chance of accompanying complications is also a major treatment goal in transplant patients. It is recommended to initiate administration of antivirals in patients with HZ within 72 h after cutaneous symptoms onset, or at a later time. Administration as early as possible can decrease the intensity and duration of zoster-associated pain, prevent dissemination and fulminant visceral involvement, accelerate the healing of skin lesions, and improve the affected patients’ quality of life. Antiviral agents can still be administered in certain patients who present >72 h after the onset of skin symptoms. These patients include elderly patients over 60 years of age with severe pain and HZ in large affected skin areas, patients with persistent new vesicle formation, or immunocompromised patients, and in those with complicated HZ (28). Antiviral therapy can be stopped when no more new vesicles appear. If vesicle formation sustains during treatment over 7 days, the diagnosis should be re-evaluated, and drug resistance may be a problem (40).

The synthetic nucleoside analog of guanine acyclovir and its prodrug valacyclovir, penciclovir’s prodrug famciclovir, and the thymidine analog brivudin are the most widely used drugs for the HZ antiviral treatment (1). When choosing an antiviral drug, all possible factors influencing efficacy should be considered, including routes of administration, dosage frequency, costs, complications contraindications, and drug interactions (40).

#### Acyclovir

Acyclovir was the first drug discovered that had antiviral activity against herpes virus and is very safe and well tolerated (41). HZ in immunocompetent patients can be treated orally, but due to the poor oral bioavailability (15–30%) of acyclovir, other simpler dosing and better pharmacokinetic antiviral drugs (valacyclovir or famciclovir) are preferred (1, 42). Intravenous acyclovir with 500 mg/m^2^ every 8 h is the initial therapy of choice for a varicella-like rash in SCT recipients. When the infection is controlled, oral antiviral medication is an option for the remainder of the treatment (43).

#### Valacyclovir

Valacyclovir is a prodrug of acyclovir with three to five times higher oral bioavailability than acyclovir (44), and it was approved for use as an additional treatment for herpes virus infections in the USA in 1995 (45). The convenient dosing schedule (1,000 mg three times daily) and quicker cessation of pain makes valacyclovir more efficacious than acyclovir in treating acute HZ (46).

#### Famciclovir

Famciclovir is a well-absorbed (bioavailability 77%) first-line option for the treatment of HZ and used for treating HZ in immunocompetent adults and immunosuppressed patients older than 25 years, but not approved in childhood and adolescence (1). In a study with 148 patients who were immunocompromised following bone marrow or solid organ transplantation or oncology treatment, the efficacy and safety of famciclovir was evaluated. The study showed that oral famciclovir is convenient, effective, and well tolerated for immunocompromised patients with HZ (47).

#### Brivudin

Brivudin is a thymidine nucleoside analog with stronger antiviral effect against VZV than reference compounds such as acyclovir by blocking the action of DNA polymerases. Oral brivudin (125 mg once daily) is licensed for the treatment of HZ in several countries of the European Union (48). In a double-blind randomized multicenter study, 48 immunocompromised patients with a HZ rash less than 72 h in duration received brivudin or intravenous acyclovir treatment. No significant difference was seen regarding cutaneous or visceral dissemination compared between the two therapies (49). Brivudin is not available in all countries and not approved for antiviral therapy in children and adolescents due to lacking of studies about safety profile (1, 40).

#### Antiviral Agents Used to Treat Herpes Viruses Other Than VZV and Co-Infections

Ganciclovir and its valyl-ester valganciclovir, and foscarnet are primarily used to treat cytomegalovirus (CMV) infections (50). Both agents are nevertheless active against VZV and may be used to treat double infections or to suppress reactivations (51).

Although foscarnet dosing to treat VZV has not been studied extensively, doses required to treat CMV infections have been shown to treat VZV successfully. Foscarnet, however, is considerably more toxic than acyclovir. It is only recommended for the treatment of CMV co-infections, or when antiviral resistance is suspected (52).

Likewise, ganciclovir and valganciclovir are largely used for the treatment of CMV infections, but both agents possess excellent activity against VZV in doses used to treat CMV infections (51). Its toxicity especially to bone marrow restricts its use to prophylaxis and treatment of CMV co-infections (53, 54).

### Postherpetic Neuralgia

Postherpetic neuralgia tends to be underdiagnosed and inadequately managed, so treatments of PHN are palliative and shortening of duration and severity of pain is the main goal of PHN management (55, 56). According to the guidelines (2010) issued by the European Federation of Neurological Societies, tricyclic antidepressants (TCAs), antiepileptics (gabapentin/pregabalin), and topical lidocaine plaster (5%) are recommended as first-line treatment in PHN (57).

#### Tricyclic Antidepressants

Tricyclic antidepressants, including two classes—the secondary amines (nortriptyline and desipramine) and the tertiary amines (amitriptyline and imipramine)—have been commonly used for the treatment of PHN from the early 1980s (55). TCAs can block voltage-dependent sodium channels and α-adrenergic receptors and also inhibit the reuptake of monoaminergic transmitters which can enhance the effects of biogenic amines in modulating descending pain pathways (58). Clinical experience showed TCAs to be effective in the control of PHN and amitriptyline is the most commonly used TCAs which can improve patients sleep more due to its sedating property. However, TCAs are often poorly tolerated with a relatively slow onset of action and associated with systemic adverse events (AEs) such as dry mouth, constipation, serotonin syndrome, cardiotoxicities, and anticholinergic effects. TCAs side effects are common in the mostly elderly population afflicted with PHN thus alternative treatments may be considered (3).

#### Antiepileptics

Oral antiepileptics gabapentin and pregabalin can block voltage-sensitive calcium channel, decrease the release of several neurotransmitters, and inhibit central pain pathways. In clinical trials, gabapentin and pregabalin significantly reduced PHN-related pain and improved sleep quality more than placebo (59, 60). Gabapentin may be superior to other formulations in terms of compliance and safety, while pregabalin was associated with better health outcomes and cost-effectiveness (60, 61). The most common side effects of gabapentin and pregabalin are dizziness, somnolence, and peripheral edema (62, 63). These AEs could limit their use in some patients.

#### Lidocaine Plasters (5%)

Lidocaine-medicated plasters (LMP) were approved to use in the treatment of PHN by the Food and Drug Administration (FDA) in 1999 and have been licensed in several European, Latin American, and the Middle East countries since then (64). As a topical analgesic, 5% LMP can deliver the drug directly to the site of pain and provides local pain relief through localized analgesia, without inducing anesthesia or numbness (64). In a systematic review including 20 unique studies, 5% LMP showed similar effects on pain relief compared with gabapentin and was more effective than placebo, capsaicin, and pregabalin (change in pain from baseline) (65). 5% LMP is more cost-effective than pregabalin in a UK setting (66) and demonstrated good short- and long-term tolerability with a minimal risk for systemic adverse drug reactions (67).

#### Polypharmacy

Because monotherapy may not be sufficient of modifying all of the complex pain mechanisms that underlie PHN, combination therapies are frequently applied and five or more drugs on average are taken by a typical PHN patient (56). Due to the low tendency of gabapertinoids and topical analgesics for interactions with other drugs, they have become the most attractive agents for combination therapies (55). A randomized, open-label, multicenter, non-inferiority study indicated that combination of pregabalin and 5% LMP provided additional efficacy for pain relief in PHN in patients unresponsive to either monotherapy (68).

Concerning transplant patients, their complicated condition and weak organ function such as renal impairment, could affect drug metabolism and tolerability (69). In the setting of polypharmacy, it would be more difficult to choose and dosage would also change according to every patient’s situation, while side effects of different drugs should also be considered. Besides, once patients develop PHN after HZ, the condition does not usually adequately respond to treatment (70).

## Prevention/Prophylaxis

### Vaccination

Current treatment strategies for HZ and PHN are only partially effective, so reducing the burden of HZ by prophylactic vaccination may be a proactive strategy (71).

#### Live-Attenuated Zostavax^®^ (Merck, USA)

In 2006, the FDA approved the 1-dose live-attenuated HZ vaccine Zostavax^®^ for use in individuals 60 years of age and older and expanded the use of Zostavax^®^ among adults aged 50 through 59 years in March 2011 (72). In 2017, the Advisory Committee on Immunization Practices (ACIP) reviewed their guidelines and Zostavax^®^ remained as a recommended vaccine for immunocompetent adults aged over 60 years to prevent HZ (73).

Zostavax^®^ contains at least a 14 times higher titer of the same live-attenuated Oka/Merck strain used in the varicella vaccine (Varivax^®^, a live-attenuated varicella vaccine for the prevention of chickenpox) and is therefore only recommended for VZV seropositive people (74). Several studies have shown the efficacy of Zostavax^®^ in HZ and PHN prevention. The Shingles Prevention Study (SPS) was a randomized, double-blind, placebo-controlled study that enrolled 38,546 adults over the age of 60 years and showed that Zostavax^®^, respectively, reduced the incidence of HZ and PHN by 51.3 and 66.5% in 4 years of postvaccination follow-up. However, the efficacy against HZ incidence decreased with age (37.6% among subjects ≥70 years old and 63.9% among younger subjects) (75, 76). To further assess the persistence of vaccine efficacy, the Short-Term and Long-Term Persistence Substudy were undertaken following SPS. The STPS re-enrolled 14,270 SPS vaccine and placebo recipients, and after 5 years, vaccine efficacies for HZ burden of illness, the incidence of PHN, and the incidence of HZ decreased from 61.1 to 50.1%, 66.5 to 60.1%, and 51.3 to 39.6%, respectively (77). In the LTPS, vaccine efficacy declined for all these three outcome measures from 7 to 11 years postvaccination (78). To reverse this decline in efficacy, a booster dose of Zostavax^®^ could be a potential solution. HZ vaccine was given as a second dose to 200 subjects over the age of 70 years who had received one dose Zostavax^®^ ≥10 years before in a study, and the VZV-specific cellular and humoral responses to this booster dose were compared with older and younger vaccinee. This study showed that while VZV antibody levels were similar in all age groups, VZV-specific CMI was significantly higher in the booster-dose group. The authors suggested that more clinical trials of appropriate age of vaccine administration as well as the potential of booster dose would be required (79).

Because post-transplantation, humoral and cellular responses to vaccines are suboptimal, it is important to immunize patients while they are awaiting transplantation. This should optimally be part of a comprehensive pre-transplant evaluation and preparation (80). Zostavax^®^ is contraindicated after transplantation, because of the increased risk for live vaccine-induced infections, caused by the altered immunocompetence. According to the 2008 ACIP guideline, zoster vaccine should be administered at least 14 days before initiation of immunosuppressive therapy. Zoster vaccine is recommended in HSCT candidates over 4 weeks before transplantation but contraindicated after HSCT due to limited experience of HSCT recipients with life VZV-containing vaccines (14). Vaccination may be considered after assessing of the immune status of the recipient on a case-by-case basis and only administered at least 24 months after HSCT (81).

A study retrospectively assessed the use of Zostavax^®^ in 62 patients with hematologic malignancy. Among them, 31 patients received hematopoietic cell transplant (HCT), and the median times to vaccination post-transplant were 482 days (26 patients received autologous HCT) and 1,323 days (5 patients received allogeneic HCT). There were no documented AEs associated with administration of Zostavax^®^ among all the patients. One patient developed HZ 3 weeks after vaccination, but they could not establish whether this was caused by wild-type VZV or the Oka/Merck strain because genotyping data were not included in this study (82). In another study, a single dose of Zostavax^®^ was given to 110 adult autologous and allogeneic HSCT recipients about 2 years post-transplantation. Two patients developed a skin rash with unclear reason after vaccination and 98.2% of vaccine recipients had no AEs within a median 9.5 months follow-up period (83). In a retrospective study about the safety of measles–mumps–rubella (MMR) vaccine and Zostavax^®^ in multiple myeloma patients about 24 months after autologous HCT, 70 patients on maintenance lenalidomide received one dose HZ vaccine (69 patients also received MMR vaccine). No rash or other AEs related to the vaccines were identified and one patient with previous PHN history experienced worsening pain without rash while the reason leading to this situation was unknown (84). Miller et al. conducted a randomized, placebo-controlled study, which enrolled 34 participants with end-stage renal disease awaiting renal transplantation (26 subjects received one dose Zostavax^®^ and 8 received placebo). From 30 to 235 days after vaccination, 14 subjects underwent transplantation and 12 of them received zoster vaccine. There were no zoster rashes or reactogenicity events associated with vaccine that happened to subjects (85). Though limitations exists, including small sample size and lacking of longer follow-up, these studies show a potential benefit of HZ vaccine in transplant patients before and after the transplantation with a good safety profile. Larger, controlled studies are needed to further explore the safety and efficacy of Zostavax^®^ in HCT and SOT population (82, 83).

#### The HZ Subunit Vaccine (HZ/su, Shingrix^®^)

Subunit vaccines can provoke a strong immune response, while being a safe vaccine for transplant patients, because they avoid the risk of disease caused by replication of the vaccine virus (86).

A new recombinant subunit vaccine (Shingrix^®^) containing VZV gE and the AS01B adjuvant system was tested by GlaxoSmithKline in 2015. In a phase III randomized study including 15,411 participants (≥50 years of age) from 18 countries, two doses of the HZ/su administered 2 months apart had a vaccine efficacy of 97.2% and significantly reduced the HZ in adults 50 years of age or older (87). HZ/su also had a vaccine efficacy of 89.8% in adults over 70 years old and showed no decline in efficacy with age (88). Besides, a follow-up, multicenter, single group trial conducted in the Czech Republic, the Netherlands, Germany, and Sweden indicated that both gE-specific cellular and humoral immune responses decreased over time but remained substantially above prevaccination levels for up to 6 years in healthy older adults who had received two doses of HZ/su. HZ/su seems to have the potential to provide long-term protection against HZ in older adults (89). Solicited reports of injection-site and systemic reactions such as fatigue and myalgia were more among HZ/su than placebo, but the reactions were generally mild-to-moderate in intensity and serious AEs and deaths were similar in the vaccine and placebo groups (87–89). A cost-effectiveness analysis using Markov decision model compared HZ subunit vaccine, Zostavax^®^ or no vaccination in immunocompetent adults ≥60 years old. The schedule with two doses HZ/su (at a price of $280 per series) was found to be more effective and at lower costs than Zostavax^®^ (90). Shingrix^®^ was approved by FDA for the prevention of HZ in adults older than 50 years old and recommended by ACIP for use in immunocompetent adults aged ≥50 years in October 2017. ACIP also made the comment that Shingrix^®^ is recommended for immunocompetent adults who received live-attenuated HZ vaccine before and Shingrix^®^ is preferred over Zostavax^®^ for the prevention of HZ and related complications (73).

With regard to transplant patients, so far there is only one published study about the HZ/su in autologous HCT recipients. In this phase I/II, randomized, observer-blind, placebo-controlled study, three-dose regimen was found well tolerated and immunogenic in adults who had undergone autologous HSCT 50–70 days earlier. Both humoral and cellular immune responses were strong and persisted for up to 1 year postvaccination. Although with annotated limitations of small numbers of subjects and requirements for additional follow-up, the result shows a promising prospect for immunization of transplant patients (91). More studies especially phase III follow-up trials are still needed. Finally, a phase III trial assessing the efficacy of HZ/su in autologous stem cell transplant patient is under way and phase I/II trials in SOTs are in progress (92).

### Antiviral Agents

Long-term acyclovir/valacyclovir prophylaxis to prevent recurrent VZV infection is routinely recommended by the 2009 International Guidelines for the first year after HCT for VZV-seropositive allogeneic (should generally be offered) and autologous (optional) HCT recipients (93). In a systematic review including six observational studies involving 3,420 HSCT recipients, the optimal duration of antiviral prophylaxis (acyclovir, valacyclovir, and famciclovir) for preventing HZ was analyzed. It was found that antiviral prophylaxis may significantly decrease the reactivation of VZV in HSCT recipients and patients taken antiviral prophylaxis at least 1 year compared with less than 1 year showed lower incidence of HZ (2.1 and 15.4%, respectively) (94). In SOT recipients, prophylaxis such as acyclovir has not been systematically studied (26). Koo et al. reviewed the medical files of 314 heart transplant recipients from 1995 to 2010. 134 patients received ganciclovir/valganciclovir-based CMV prophylaxis for a median of 143 days post-transplantation. The majority of patients develop VZV infection during the first post-transplant year and the risk of HZ decreased with ganciclovir or valganciclovir prophylaxis (25). In another study including 444 adult kidney transplants, administering anti-CMV prophylaxis (ganciclovir/valganciclovir) for 3–6 months after transplantation reduced the risk of HZ and no patients developed HZ during CMV antiviral prophylaxis (20). Although antiviral prophylaxis is effective for preventing HZ, the risk of VZV reactivation is still much higher in transplant patients than the general population (20, 95, 96). It is also difficult to completely prevent the development of HZ after the discontinuation of prophylaxis. Once prophylaxis was discontinued, the cumulative risk for developing VZV disease remained high (95, 97). Besides, the emergence of resistant VZV strains due to long-term prophylaxis is also a potential risk that cannot be neglected despite no antiviral agent-resistant VZV was observed so far (97).

### Post-Exposure Prophylaxis (Seronegative Patients Only)

Varicella-zoster immune globulin (VZIG) is a purified human IgG with high titers of antibodies to VZV and used to provide passive immunization for seronegative patients exposed to VZV (98). In December 2012, FDA approved a VZV immune globulin preparation VariZIG (Cangene Corp., Winnipeg, MB, Canada) for use in the U.S. as a post-exposure prophylaxis of varicella (99). In July 2013, the Centers for Disease Control and Prevention (CDC) updated recommendations for use of VariZIG. The CDC recommended that patients without evidence of immunity to varicella who are at high risk for severe varicella and complications should receive VariZIG as soon as possible after exposure to varicella-zoster virus and within 10 days. Patient groups recommended by CDC to receive VariZIG include the following:

(1) immunocompromised patients without evidence of immunity;
(2) newborn infants whose mothers have signs and symptoms of varicella around the time of delivery (i.e., 5 days before to 2 days after);
(3) hospitalized premature infants born at ≥28 weeks of gestation whose mothers do not have evidence of immunity to varicella;
(4) hospitalized premature infants born at <28 weeks of gestation or who weigh ≤1,000 g at birth, regardless of their mothers’ evidence of immunity to varicella; and
(5) pregnant women without evidence of immunity (99).

So far, there is a lack of evidence in prior studies about the use of VZIG to prevent severe varicella, HZ, and relevant complications in SOT patients, and most reports about successful use of VZIG are in pregnant women and infants. In a study form Denmark involving 104 pregnant women who received VZIG from December 2005 to March 2015, only five (6%) women developed varicella during VZIG treatment and VZIG seems effective in preventing varicella and zoster (100). In a retrospective record review of 812 adult renal transplant recipients, performed from 1995 until 2004 in a single center, eight patients developed varicella infection and six of these patients received VZIG with acyclovir. There was no significant impact of the use of VZIG on the clinical spectrum of the disease and the authors concluded that passive immunization with VZIG is useless once clinical varicella has already established, a finding that is in agreement with other studies (101). Due to the high cost and short supply of VZIG in some, alternative approach of prophylaxis using antiviral agents or vaccination against VZV is needed (43, 102, 103).

## Prospect

Despite great advances in transplantation in recent decades, infection still is a major cause of morbidity and mortality among transplant recipients. Transplant patients are at high risk for developing HZ and accompanying complication such as PHN and disseminated cutaneous disease (14, 18, 104). In addition, treatments for transplant patients are more difficult because of their complicated conditions and reduced organ function (69). Regular screening to evaluate each patient’s humoral and cellular immunity against VZV and ensuring early recognition and preventing HZ by vaccination may be a proactive strategy. Currently, there are two licensed HZ vaccine, the live-attenuated vaccine Zostavax^®^ and the recombinant subunit vaccine Shingrix^®^. For immunocompetent older people within the recommend immunization age, vaccination rate is still low in the U.S. In a retrospective observational study conducted in 2015, 6,746,476 U.S. adults aged ≥60 years during 2007–2013 and 6,770,294 adults aged 50–59 years during 2011–2013 were identified as vaccinated. This study found that 19.5% of adults aged ≥60 years received an HZ vaccine, which is lower than the 30% target of Healthy People 2020 (including objectives designed to serve as this decade framework for improving the health of all people in the U.S.) (105). For transplant patients, HZ vaccine usage after transplantation is still contraindicated in this population due to its live-attenuated characteristic. Pre-transplantation immunization is considered a relevant alternative, but efficacy of protection after transplantation still needs to be established. Both HZ vaccines have been tested in autologous HCT recipients and showed promising safety and efficacy (Table 2). Shingrix^®^ is a candidate for a post-transplant vaccine as it does not contain any live virus. Larger, prospective, controlled studies are warranted to further determine the safety and efficacy of Zostavax^®^ and Shingrix^®^ in transplant populations, and extra efforts are needed to increase vaccine usage according to guidelines.

**Table 2:** Herpes zoster (HZ) vaccine usage in transplant patients.

Reference	Vaccine	Number and type of patients	Median time to vaccination pre/post-transplantation	HZ after vaccination	Adverse events (AEs)
(82)	Zostavax^®^	62 patients with hematologic malignancy: among them 26 (41.9%) underwent autologous hematopoietic cell transplant (HCT); 5 (8.1%) underwent allogeneic HCT; 31 (50%) without HCT	482 days after autologous HCT; 1,323 days after allogeneic HCT	One patient underwent autologous HCT developed HZ 3 weeks after vaccination and was treated with 10 days of high dose acyclovir	No documented AEs

(83)	Zostavax^®^	52 patients underwent autologous hematopoietic stem cell transplant (HSCT); 58 patients underwent allogeneic HSCT	27 months after HSCT	One patient underwent autologous HSCT developed a skin rash 10 days after vaccination. One patient underwent allogeneic HSCT developed a vesicular skin rash 24 days after vaccination and was treated with valacyclovir	108 (98.2%) patients had no clinically apparent AEs

(84)	Zostavax^®^	70 multiple myeloma patients on maintenance lenalidomide with autologous HCT: among them 69 patients also received measles–mumps–rubella vaccination	25 months after HCT	Two patients developed a non-specific rash requiring no therapy and resolved by themselves	Upper respiratory tract infection is the most common AE (10/70 patients) One patient with pre-existing postherpetic neuralgia (PHN) experiencing worsening of PHN without development of a rash

(85)	Zostavax^®^	26 patients with end-stage renal disease awaiting renal transplantation: among them 12 (46%) received transplantation	From 30 to 235 days before transplantation	No zoster rashes happened	Local reactogenicity symptoms occurred in 9 subjects (35%). No AEs associated with vaccine

(91)	Recombinant HZ vaccine	120 patients underwent autologous HCT: 30 patients received 3 doses of gE/AS01_B_; 29 patients received 3 doses of gE/AS01_E_; 31 patients received 2 doses of gE/AS01_B_; 30 patients received 3 doses of saline	Patients underwent HCT in the previous 50–70 days	Two patients in the 3 doses of gE/AS01E and two patients in the saline group developed HZ	Most subjects experienced solicited local and general reactions of mild or moderate intensity One patient in the 2 doses of gE/AS01B group had serious AE (pneumonia) and was considered vaccine related

## Author Contributions

LW, JW, and NB contributed to the conception, design, and writing this article. EV, CL-B, SB, and AJ contributed to the revision of this article.

## Conflict of Interest Statement

The authors declare that the research was conducted in the absence of any commercial or financial relationships that could be construed as a potential conflict of interest.

## Abbreviations

ACIP, Advisory Committee on Immunization Practices; AEs, adverse events; CDC, Centers for Disease Control and Prevention; CMI, cell-mediated immunity; CMV, cytomegalovirus; CSF, cerebrospinal fluid; DFA, direct immunofluorescent assay; ELISA, enzyme-linked immunosorbent assays; ESRD, end-stage renal disease; FAMA, fluorescent antibody to membrane antigen; FDA, Food and Drug Administration; gE, glycoprotein E; HCT, hematopoietic cell transplant; HSCT, hematopoietic stem cell transplant; HSV, herpes simplex virus; HZ, herpes zoster; HZ/su, herpes zoster subunit vaccine; IFNs, interferons; IgG, immune globulin G; JAK/STAT, Janus kinase-signal transducer and activator of transcription; JNK, C-Jun N-terminal kinase; LMP, lidocaine-medicated plasters; MMR, measles–mumps–rubella; PHN, postherpetic neuralgia; SOT, solid organ transplant; SPS, Shingles Prevention Study; STAT3, signal transducer and activator of transcription 3; STPS/LTPS, Short-Term and Long-Term Persistence Substudy; TCAs, tricyclic antidepressants; TK, thymidine kinase; VZV, varicella zoster virus; VZIG, varicella-zoster immune globulin.
